# A Review on the Evolution of Thermal and Environmental Barrier Coating Systems and Their High-Temperature Degradation Mechanisms in Advanced Aero-Engines

**DOI:** 10.3390/ma19112413

**Published:** 2026-06-05

**Authors:** Saijun Ren, Yukang Sun, Han Yan, Xuyang Zhang, Yiwang Bao, Kuilin Lv

**Affiliations:** 1 China Testing & Certication International Group Co., Ltd., Chaoyang District, Beijing 100024, China; 2 School of Civil and Transportation Engineering, Beijing University of Civil Engineering and Architecture, Beijing 102616, China

**Keywords:** thermal barrier coating (TBC), CMAS corrosion, water-oxygen corrosion, failure mechanisms

## Abstract

With the continuous advancement of thrust-to-weight ratios in modern aero-engines, turbine inlet temperatures have reached levels that far exceed the thermal endurance limits of conventional superalloys and emerging ceramic matrix composites (CMCs). Consequently, thermal barrier coatings (TBCs) and environmental barrier coatings (EBCs) have become indispensable multifunctional systems for hot-section component protection. This review systematically delineates the evolutionary trajectory of TBC/EBC systems, transitioning from traditional yttria-stabilized zirconia (YSZ) and simple silicates to advanced multi-rare-earth-doped oxides, A_2_B_2_O_7_ pyrochlore structures, and high-entropy ceramic systems. A critical comparative assessment is provided regarding their phase stability, thermal-physical properties, and durability challenges above 1200 °C. Furthermore, this paper provides an in-depth analysis of high-temperature degradation mechanisms, focusing on the thermochemical and thermomechanical interactions under calcium-magnesium-alumino-silicate (CMAS) attack, water-oxygen corrosion, and molten salt infiltration. By synthesizing current research gaps, we highlight the trade-offs between low thermal conductivity, high toughness, and environmental resistance. Finally, a strategic roadmap for next-generation coatings is proposed, emphasizing the integration of high-entropy material design, multi-scale structural optimization, and AI-driven life prediction models to meet the stringent reliability requirements of future propulsion systems.

## 1. Introduction

With the continuous advancement of aero-engines and industrial gas turbines toward higher thrust-to-weight ratios and elevated turbine inlet temperatures, the thermal load imposed on hot-section components has exceeded the intrinsic limits of conventional superalloys. Thermal barrier coatings (TBCs), which consist of low-thermal-conductivity ceramic topcoats deposited on metallic substrates, have been widely adopted as the most mature solution for thermal protection [1]. To further increase operating temperatures beyond 1500 °C, lightweight and high-temperature-resistant ceramic matrix composites (CMCs) have emerged as promising alternatives to conventional metallic materials [2]. However, CMCs are highly susceptible to volatilization and degradation in high-temperature water vapor environments, which severely compromises their structural integrity. This challenge has led to the development of environmental barrier coatings (EBCs), which serve as critical protective layers for CMC components [3,4].

Despite their distinct functional roles, both TBCs and EBCs are subjected to severe degradation under complex service environments. Water vapor corrosion plays a dominant role in interfacial degradation, particularly through accelerated growth of thermally grown oxides (TGOs) and volatilization of ceramic constituents, thereby reducing interfacial durability [5]. In addition, calcium–magnesium–aluminosilicate (CMAS), originating from ingested sand, volcanic ash, and industrial particulates, has emerged as a critical factor leading to premature coating failure [6]. Molten CMAS can infiltrate porous ceramic layers, inducing stress accumulation upon solidification, while simultaneously triggering thermochemical reactions that result in phase transformation and microstructural degradation [7]. Although extensive efforts have been devoted to understanding individual degradation mechanisms in TBCs and EBCs, the coupled effects of CMAS attack and water vapor corrosion under multi-field conditions remain insufficiently understood. This lack of systematic understanding hinders the rational design of next-generation coating systems with enhanced durability. In this context, this review provides a comprehensive overview of recent advances in TBC and EBC systems for high-temperature applications. The material systems and structural characteristics of these coatings are first compared, followed by a detailed discussion on interfacial degradation induced by water vapor corrosion and CMAS attack [8]. Furthermore, the synergistic effects of these degradation modes are analyzed, and current strategies for improving corrosion resistance are critically evaluated. Finally, future directions for the design of high-performance, long-life thermal/environmental barrier coating systems are proposed.

## 2. Advances in Thermal Barrier Coatings

**Figure 1:**
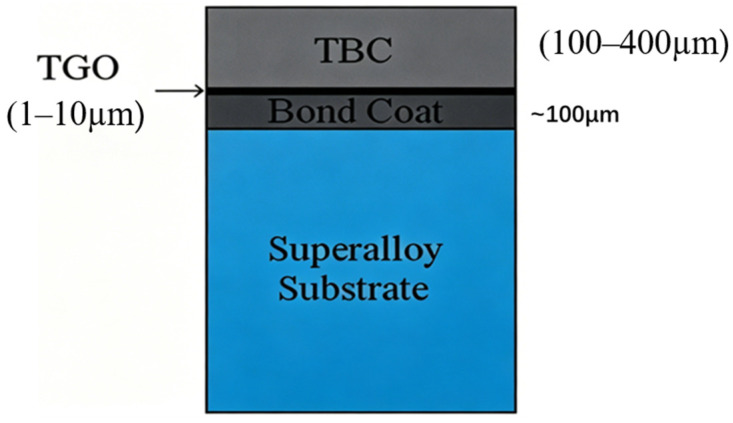
Typical multi-layer architecture of a TBC system, highlighting the strategic arrangement of the ceramic topcoat (TC), thermally grown oxide (TGO), metallic bond coat (BC), and superalloy substrate for thermal protection.

As the thrust-to-weight ratio of aero-engines continues to increase, the temperature and pressure within combustion chambers have reached extreme levels, leading to turbine inlet temperatures of 1538–1871 °C, far exceeding the service limits of existing materials. The native oxide films (e.g., NiO) formed on conventional nickel-based and cobalt-based superalloys exhibit loose microstructures, significant thermal expansion mismatch with the substrate, and limited adhesion stability. As a result, they fail to provide effective protection under ultra-high-temperature oxidation and molten salt hot corrosion environments containing sulfur, vanadium, and other impurities [9]. Although single-crystal superalloys and advanced film cooling technologies have been widely implemented, their effectiveness remains constrained. The former has approached a theoretical temperature limit of approximately 1150 °C, while the latter, despite reducing surface temperatures by about 400 °C [10], compromises engine efficiency due to cooling air consumption and weakens mechanical integrity because of complex cooling hole structures [11]. Consequently, thermal barrier coating (TBC) systems, typically consisting of a ceramic topcoat (TC) and a metallic bond coat (BC), have become indispensable for high-temperature protection. As illustrated in Figure 1, a complete TBC system comprises a superalloy substrate, a bond coat, a thermally grown oxide (TGO) layer, and the ceramic topcoat (TBC) (Figure 1). The bond coat reacts with inward-diffusing oxygen to form a dense, continuous, and adherent α-Al_2_O_3_ thermally grown oxide (TGO) layer, which provides effective resistance against oxidation and hot corrosion [12]. Meanwhile, the ceramic topcoat, owing to its low thermal conductivity, significantly reduces the surface temperature of components by approximately 50–150 °C [13]. This enables the underlying alloy to operate within a safe temperature range, thereby bridging the gap between intrinsic material limits and extreme service conditions.

### 2.1. Ceramic Topcoats of TBCs

As the outermost layer directly exposed to high-temperature combustion environments, ceramic topcoats serve as the primary thermal insulation barrier in TBC systems. By virtue of their extremely low thermal conductivity, they establish a substantial temperature gradient across the coating, typically reducing the service temperature of metallic substrates by 100–300 °C or more, thereby enhancing engine efficiency and thrust-to-weight ratio [1].

To withstand the complex and aggressive service conditions of aero-engines, topcoat materials must satisfy several critical requirements. Low thermal conductivity is the foremost criterion, which can be achieved through complex crystal structures or the introduction of point defects that enhance phonon scattering and suppress lattice thermal transport [14]. Equally important is the compatibility of the coefficient of thermal expansion (CTE), as mismatch-induced stresses during thermal cycling can lead to premature cracking or spallation [15]. In addition, excellent phase stability at high temperatures is essential to avoid deleterious phase transformations accompanied by volume changes. Strong resistance to sintering is also required to preserve the porous microstructure, which is crucial for maintaining low elastic modulus and thermal insulation performance [16].

As turbine inlet temperatures continue to rise beyond 1600 °C, conventional coating systems are increasingly challenged by phase instability, accelerated sintering, and inadequate resistance to CMAS attack [8]. Consequently, the development of ceramic topcoats has evolved from single-component systems to complex multi-component compositions, and from empirical optimization toward design strategies guided by defect chemistry and thermodynamics [17]. This section therefore focuses on the evolution of advanced topcoat materials and provides a framework for the rational design of next-generation high-performance TBC systems.

#### 2.1.1. Yttrium-Stabilized Zirconia

Currently, 7–8 wt.% yttria-stabilized zirconia (7YSZ) is widely employed as the state-of-the-art ceramic topcoat material in industrial TBC systems [9]. Its widespread application originates from the unique polymorphic phase transformation behavior of zirconia. Pure zirconia undergoes a tetragonal (t) to monoclinic (m) phase transformation during cooling, accompanied by a volume expansion of approximately 3–5%, which inevitably leads to coating cracking and failure. The addition of yttria effectively stabilizes the high-temperature phases and suppresses this detrimental transformation. It has been demonstrated that a yttria content of 7–8 wt.% lies within an optimal compositional range that promotes a diffusionless martensitic transformation. During rapid cooling, the high-temperature cubic phase transforms into a metastable tetragonal t′ phase with nanoscale twin structures, rather than the monoclinic phase. Unlike the conventional tetragonal phase, the t′ phase possesses a ferroelastic twin substructure, in which twin boundaries effectively inhibit the growth of transformation products. As a result, the t′ phase exhibits excellent phase stability and remains resistant to decomposition during long-term service below 1200 °C, thereby avoiding abrupt internal stress changes associated with phase transformation-induced volume expansion [18,19]. In addition, 7YSZ exhibits a unique toughening mechanism governed by ferroelastic domain switching. Under external stress, ferroelastic domains undergo 90° reorientation, which absorbs significant crack-tip energy and induces pseudo-plastic deformation behavior. This mechanism markedly enhances fracture toughness and strain tolerance, enabling the coating to withstand severe thermal shock conditions [1,20,21]. The low thermal conductivity of 7YSZ is primarily attributed to strong phonon scattering induced by a high concentration of point defects. These defects include substitutional Y^3+^ ions replacing Zr^4+^ and the associated oxygen vacancies required for charge compensation. Consequently, the phonon mean free path is significantly reduced, resulting in a thermal conductivity as low as ~2.3 W/(m·K). Meanwhile, 7YSZ exhibits a coefficient of thermal expansion (10.5–11.5 × 10^−6^ K^−1^) that is well matched with nickel-based superalloys and MCrAlY bond coats. This compatibility minimizes thermomechanical stresses arising from thermal mismatch and reduces crack initiation at the thermally grown oxide (TGO) interface, thereby extending coating lifetime [1,19,22].

However, the applicability of conventional YSZ is fundamentally limited at temperatures exceeding 1200 °C. Accelerated atomic diffusion at elevated temperatures leads to progressive sintering, resulting in pore closure, increased thermal conductivity, and reduced strain tolerance. Simultaneously, the metastable t′ phase gradually decomposes into equilibrium phases, and subsequent cooling induces t–m transformation accompanied by volume expansion, ultimately causing coating degradation [23,24].

In addition, CMAS attack poses a critical challenge to YSZ-based coatings. Molten CMAS can readily infiltrate the porous structure of the ceramic layer, leading to densification and loss of strain compliance. Upon solidification, the infiltrated CMAS forms a rigid glassy phase that impedes stress relaxation during thermal cycling. More critically, CMAS reacts chemically with YSZ by dissolving yttria stabilizers, thereby destabilizing the t′ phase and promoting t–m transformation during cooling, accompanied by significant volume expansion [25]. These combined effects ultimately result in severe spallation failure. These intrinsic limitations of YSZ at elevated temperatures have driven the development of next-generation TBC materials with improved phase stability, enhanced sintering resistance, lower thermal conductivity, and superior resistance to CMAS attack and environmental degradation.

#### 2.1.2. Multi-Rare-Earth-Doped Systems

**Figure 2:**
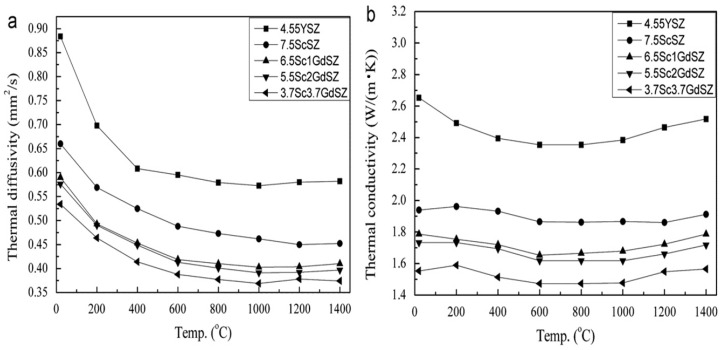
Temperature-dependent (**a**) thermal diffusivity and (**b**) thermal conductivity of various Sc/Gd co-doped zirconia systems compared with 4.5YSZ, demonstrating the substantial reduction in heat transport achieved through multi-rare-earth doping [27].

Multi-rare-earth doping, which introduces various aliovalent rare-earth cations into the ZrO_2_ lattice, not only induces strong phonon scattering through severe lattice distortion and high concentrations of oxygen vacancies, thereby significantly reducing the lattice thermal conductivity [26], but, more importantly, increases the configurational entropy of the system to stabilize the metastable t′ phase, effectively suppressing the irreversible t → m phase transformation above 1300 °C that leads to delamination failure. In the synergistic regulation of thermal insulation performance and phase stability, studies have shown that systems such as Sc/Gd [27], Gd/Yb [28], and Gd/Yb/Y [29] exhibit significant advantages. For example, compared with conventional YSZ, the thermal conductivity of 3.7Sc3.7GdSZ is reduced by approximately 40% (Figure 2) [27], while maintaining excellent structural integrity at 1400 °C.

However, evaluating the merits of novel coating systems cannot be limited to static thermophysical indicators; it is essential to assess their durability under complex service stress fields. Sadowski et al. [30] pointed out that the core value of a thermal barrier coating (TBC) system lies not only in thermal insulation but also in its ability to inhibit crack initiation and propagation under thermo-mechanical cyclic loading. This resistance to progressive degradation is a key factor determining the service life of turbine blades. This implies that simply pursuing low thermal conductivity often comes at the expense of strain tolerance. For instance, the severe lattice distortion induced by doping, although increasing the hardness of the material [28], also significantly enhances the brittleness of the ceramic layer. To address this bottleneck, researchers have proposed introducing an appropriate amount of monoclinic phase to form a “composite-phase toughened structure” (e.g., 4Gd-2Yb-4Y [31]), which dissipates crack tip energy through phase transformation, thereby substantially improving the mechanical reliability of the material under severe thermal cycling conditions at the macroscopic level.

Furthermore, sintering resistance and spallation resistance are core indicators for evaluating long-life coatings. When conventional YSZ is serviced above 1200 °C, it is prone to severe sintering, leading to pore closure and an increase in elastic modulus, which in turn causes thermal stress concentration and induces spallation. In comparison, the GYb-YSZ system exhibits a sintering resistance three times that of 8YSZ, effectively maintaining a high strain tolerance under extreme temperature conditions and significantly extending the service life. In addition, nanoscale design (e.g., YLaYbZr [32]) and double ceramic layer (DCL) structural optimization (e.g., YbYSZ/YSZ [33]) have also been shown to synergistically enhance environmental adaptability and thermal shock cycling life.

In summary, multi-rare-earth co-doping has achieved breakthroughs in thermophysical properties at the laboratory scale. However, when examining its engineering prospects, significant research gaps remain. First, most existing studies focus on static thermal stability [32,34], lacking dynamic evaluation of spallation mechanisms under coupled thermo-mechanical-environmental fields, especially concerning complex failure behaviors such as wrinkling spallation caused by non-uniform thermally grown oxide (TGO) growth [29]. Second, the negative impact of high-concentration doping on fracture toughness has not been fundamentally resolved. The future development logic should shift from “single-function thermal insulation optimization” to an “integrated balancing of thermal/mechanical/cycling stability”, elevating fracture toughness and sintering resistance to core design criteria equally as important as low thermal conductivity, in order to meet the stringent durability requirements of next-generation high thrust-to-weight ratio engines for coating performance.

#### 2.1.3. A_2_B_2_O_7_-Type Pyrochlore Oxides

As turbine inlet temperatures continue to exceed the operational limits of conventional YSZ (~1200 °C), the development of alternative topcoat materials with superior high-temperature stability has become imperative. Among the candidates, A_2_B_2_O_7_-type oxides (A = rare-earth elements; B = Zr, Hf, or Ce) with pyrochlore or defect fluorite structures have attracted considerable attention due to their intrinsically low thermal conductivity and high melting points.

**Figure 3:**
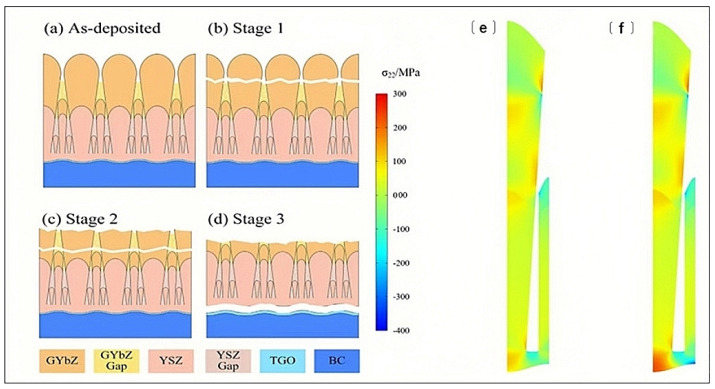
Evolution of thermomechanical failure in GYbZ/YSZ DCL coatings under PS-PVD: (**a**) as depositedstated, from initial delamination at column tips (**b**) to complete spallation at the YSZ/TGO interface (**d**), (**c**) Stage 2: Delamination of the lower part of the GYbZ columns, supported by stress distribution maps (**e**,**f**) [40].

With respect to gadolinium zirconate (GZO) and its doping-modified variants, Levi reviewed the evolutionary trend from YSZ toward pyrochlore-structured materials and pointed out that such materials strengthened phonon scattering via their complex crystal structures. Pyrochlore-type zirconates, typified by Gd_2_Zr_2_O_7_ and La_2_Zr_2_O_7_, exhibited lower thermal conductivity and superior phase stability. Nevertheless, they suffered from poor chemical compatibility with the thermally grown oxide (TGO) layer and tended to react to form secondary phases such as aluminates. In addition, their relatively low thermal expansion coefficients led to a severe thermal mismatch with metallic substrates, thus necessitating the introduction of a YSZ interlayer to relieve thermal stress and enhance bonding strength [35]. Guo et al. synthesized Yb_2_O_3_-doped Gd_2_Zr_2_O_7_ ceramics via solid-state reaction. The results showed that Yb doping induced a structural transformation from pyrochlore to fluorite, which significantly reduced thermal conductivity and increased the thermal expansion coefficient. Among them, (Gd_0.9_Yb_0.1_)_2_Zr_2_O_7_ presented the lowest thermal conductivity and favorable high-temperature phase stability in the range of 20–1600 °C [36]. Li et al. prepared quasi-columnar (Gd_0.9_Yb_0.1_)_2_Zr_2_O_7_ (GYbZ) coatings by PS-PVD. A dense reaction layer formed within one hour of contact with CMAS, which effectively prevented further infiltration of CMAS melt. The layer thickness remained nearly unchanged during thermal exposure from 1 to 100 h, demonstrating excellent CMAS corrosion resistance [37]. In terms of mechanical properties, Frommherz et al. [38] fabricated GZO/YSZ double-layer coatings through atmospheric plasma spraying (APS) and tested the delamination toughness under mixed-mode conditions. It was found that the microstructure of the GZO layer exerted a remarkable effect on delamination behavior. Coatings with high unmelted particle content and high microcrack density possessed lower toughness. In addition, stiffness measurement of free-standing GZO layers by the impulse excitation technique indicated that GZO showed better sintering resistance than YSZ above 1300 °C, with a much lower stiffness growth rate. Zhou et al. [39] prepared double-layer and triple-layer YSZ/GZO thermal barrier coatings. Their experimental results confirmed that multilayer structures achieved longer service lives than single-layer YSZ coatings under thermal cycling and CMAS erosion conditions. Li et al. [40] explored the failure behavior of quasi-columnar GYbZ/YSZ double-layer coatings fabricated by PS-PVD during burner rig tests. Combined with finite element simulation, it was concluded that coating failure was mainly dominated by interfacial delamination. Microcracks firstly initiated in the particle-embedded area beneath GYbZ column tips, then propagated downward along the columns, and eventually caused overall spallation at the YSZ/TGO interface (Figure 3). Multiple out-of-plane stress concentration points were identified, which were closely related to structural discontinuities including column tips, particle-rich zones and layered interfaces. These structural defects acted as the key driving force for delamination failure.

Lanthanum cerate (La_2_Ce_2_O_7_, LC), as a representative A_2_B_2_O_7_-type compound, has attracted increasing attention due to its excellent phase stability and resistance to high-temperature corrosion. Wang et al. [41] fabricated La_2_Ce_2_O_7_/8YSZ (LC/8YSZ) dual-layer coatings on nickel-based superalloys via EB-PVD. After cyclic corrosion at 950 °C for 100 h, the LC layer exhibited no evident phase decomposition or structural degradation. Only a limited amount of silicate compounds, such as (La,Ce)_10_(SiO_4_)_6_O_3_, formed on the surface, while the columnar microstructure remained intact. Notably, the columnar grains did not serve as fast diffusion channels for corrosive species, and the thermally grown oxide (TGO) layer maintained a dense and continuous morphology without through-thickness cracking or spallation. These results indicate that LC-based coatings possess excellent resistance to sintering and hot corrosion under aggressive environments. The microstructural evolution of LC coatings is strongly dependent on powder characteristics during deposition. Zhao et al. [42] demonstrated that powders with smaller particle size, weaker agglomeration strength, and finer particles are more readily evaporated in the plasma jet during PS-PVD, thereby promoting the formation of well-defined columnar structures. This finding highlights the critical role of feedstock design in tailoring coating architectures and performance.

Beyond single-component optimization, high-entropy ceramics have recently emerged as a novel design paradigm for next-generation thermal barrier coatings. By introducing multiple principal elements, these materials achieve configurational entropy stabilization, which promotes the formation of single-phase solid solutions and enhances structural stability at elevated temperatures. In addition, severe lattice distortion and abundant point defects significantly intensify phonon scattering, resulting in ultralow thermal conductivity.

Li et al. [43] designed defect-fluorite high-entropy oxides, among which YbGdTaHfZr exhibited an ultralow thermal conductivity of 0.61–0.89 W·m^−1^·K^−1^, while LaYYbGdTaZr demonstrated a high coefficient of thermal expansion (11.09 × 10^−6^ K^−1^ at 1400 °C), indicating excellent thermophysical compatibility. Song et al. [44] further reconstructed A_2_B_2_O_7_ and A_3_B_3_O_7_ into single-phase high-entropy fluorite structures, reducing thermal conductivity to 1.06 W·m^−1^·K^−1^. Huang et al. [45] ssynthesized single-phase fluorite high-entropy rare-earth zirconates, among which (Sm_0.2_Gd_0.2_Dy_0.2_Er_0.2_Tm_0.2_)_2_Zr_2_O_7_ exhibited a 44.6% reduction in thermal conductivity compared with YSZ without compromising stiffness.

**Figure 4:**
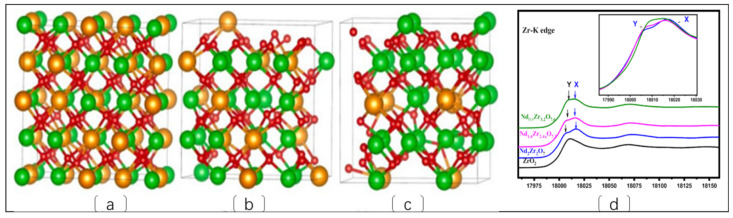
Optimized stoichiometric and non-stoichiometric pyrochlore structures from DFT calculations: (**a**) stoichiometric Nd_2_Zr_2_O_7_; (**b**) non-stoichiometric Nd_1.6_Zr_2.4_O_7.2_; (**c**) defect fluorite structure Nd_0.8_Zr_3.2_O_7.6_. Nd, Zr, and O atoms are represented by orange, green, and red spheres, respectively. XANES spectra of the Zr K-edge (**d**) [46].

Furthermore, compositional complexity and local structural disorder can be synergistically tailored to optimize thermophysical properties. Sahu et al. [46] revealed that B-site non-stoichiometry induces a transition from ordered pyrochlore to defect fluorite structures, accompanied by the formation of localized disordered regions. This “hybrid structure” (Figure 4) enhances phonon scattering and reduces lattice energy, thereby simultaneously decreasing thermal conductivity and increasing the coefficient of thermal expansion. Similarly, Li et al. [47] demonstrated that non-equimolar high-entropy design can induce phase coexistence and grain refinement, while effects such as Yb^3+^ “rattling” and oxygen vacancies further enhance phonon scattering, leading to reduced thermal conductivity.

Overall, these studies indicate that the evolution of A_2_B_2_O_7_-based materials is transitioning from conventional compositional optimization toward entropy-driven structural design. Such strategies provide a promising pathway to simultaneously achieve low thermal conductivity, high phase stability, and improved thermomechanical compatibility, thereby offering significant potential for next-generation thermal barrier coating systems.

Beyond conventional A_2_B_2_O_7_ systems, a variety of advanced ceramic materials have been explored to further enhance the multifunctional performance of thermal barrier coatings, particularly in terms of toughness, corrosion resistance, and thermal insulation. Among these, high-entropy rare-earth niobates and tantalates have attracted increasing attention. These materials typically exhibit defect fluorite structures, high Vickers hardness (10.9–12.0 GPa), moderate coefficients of thermal expansion, and excellent phase stability, along with good chemical compatibility with Al_2_O_3_, making them promising candidates for next-generation TBC applications. Rare-earth tantalates (RETaO_4_) are of particular interest due to their zirconia-like ferroelastic phase transformation toughening behavior. This mechanism enables stress-induced domain switching, which effectively dissipates crack-tip energy and mitigates the intrinsic brittleness of ceramic coatings, thereby enhancing resistance to spallation [48].

In addition to single-phase materials, multiphase composite design has emerged as an effective strategy to tailor thermophysical properties. Ren et al. [49] introduced Sr_2_Nb_2_O_7_ into 24 mol% Y_2_O_3_-stabilized HfO_2_ (YSH24), resulting in the formation of a Nb^5+^-doped SrHfO_3_ perovskite phase and a dual-phase microstructure consisting of fluorite and layered perovskite phases. This hybrid structure effectively suppressed radiative heat transfer and enhanced phonon scattering, leading to a low thermal conductivity of 1.63 W·m^−1^·K^−1^ at 1200 °C and an 86% reduction in radiative thermal conductivity. Furthermore, phosphate and aluminate-based materials have been widely employed to improve CMAS resistance and fracture toughness. Wu et al. [50] demonstrated that the incorporation of NdPO_4_ into NdYbZr_2_O_7_ ceramics increased fracture toughness by approximately 25%, which can be attributed to crack deflection and interface strengthening effects. Guo et al. [51] showed that Yb doping in GdPO_4_ reduced the formation enthalpy of apatite phases, thereby facilitating the formation of a dense and adherent reaction layer that effectively inhibits CMAS infiltration. Similarly, Wang et al. [52] prepared (Gd,Y)_3_Al_5_O_12_(GYAG) coatings via atmospheric plasma spraying, achieving a reduced thermal conductivity of 1.17 W·m^−1^·K^−1^ at 800 °C which is lower than that of conventional YSZ.

A critical bottleneck for the practical implementation of A_2_B_2_O_7_ pyrochlore-structured oxides is the trade-off between their exceptional thermal properties and their mechanical reliability. Although pyrochlore structures such as Gd_2_Zr_2_O_7_ exhibit significantly lower thermal conductivity than YSZ, their intrinsic fracture toughness is typically much lower than that of conventional YSZ. Experimental evidence shows that during severe thermal cycling, Gd_2_Zr_2_O_7_-based coatings are highly susceptible to premature spallation because they lack the necessary toughness to arrest crack propagation within the ceramic layer. Consequently, improving fracture toughness has become a critical prerequisite for the engineering application of next-generation low-thermal-conductivity ceramic layers to ensure they can survive the extreme thermomechanical loads in advanced aero-engines.

It is worth noting that, in addition to the A_2_B_2_O_7_ system, perovskite-structured oxides (e.g., LaCoO_3_) have also shown unique potential as novel high-temperature protective materials. Vasudevan et al. [53] reported that LaCoO_3_ prepared via a sol-gel method exhibits good monoclinic phase stability and a porous aggregated morphology. Although such materials are currently more commonly studied in the field of electrocatalysis, their tunable porosity during synthesis and adaptability to complex environments offer new possibilities for designing novel coatings with multifunctional integration, such as sensor functionality or active environmental protection.

In summary, A_2_B_2_O_7_-based materials and their derived high-entropy and perovskite systems have achieved significant breakthroughs in thermophysical properties at the laboratory scale. However, the core logic for future development should shift from the “pursuit of single-function metrics” back to “system robustness under service environments.” How to synergistically enhance the fracture toughness and spallation resistance of coatings through ferroelastic phase transformation toughening [48] or multiphase composite design (e.g., introducing NdPO_4_ [50]), while maintaining ultra-low thermal conductivity, will be the key step in determining whether these novel materials can transition from “literature descriptions” to “service in actual aero-engine components.”

To systematically compare the performance characteristics of different topcoat materials for thermal barrier coatings (TBCs), Table 1 summarizes the typical TBC materials covered in this study, including their thermal conductivity, coefficient of thermal expansion (CTE), typical thickness range, main advantages and disadvantages, and CMAS resistance rating. Through the comparison of conventional YSZ, rare-earth-doped systems, A_2_B_2_O_7_-type oxides, and high-entropy oxides, the differences among the various material systems in terms of thermal insulation performance, phase stability, and environmental adaptability can be analyzed more intuitively.

As can be seen from Table 1, the conventional YSZ system has a mature foundation for engineering applications, but its high-temperature sintering and CMAS resistance are limited. A_2_B_2_O_7_-type oxides and high-entropy oxides excel in reducing thermal conductivity and enhancing CMAS resistance, whereas their toughness and long-term service reliability still require optimization. Future trends in TBC material development are likely to focus on multi-component synergistic design, high-entropy formulation, and composite structure optimization.

### 2.2. Thermal Barrier Coating Metallic Bond Coat

The metallic bond coat serves as a critical interlayer in thermal barrier coating (TBC) systems, positioned between the superalloy substrate and the ceramic top coat. During high-temperature service, a dense and slow-growing α-Al_2_O_3_ thermally grown oxide (TGO) layer forms in situ on the bond coat, providing effective oxidation and corrosion resistance while inhibiting the inward diffusion of oxygen. Meanwhile, as a transition layer with a graded coefficient of thermal expansion (CTE), the bond coat significantly alleviates thermally induced stresses arising from the mismatch between the metallic substrate and the ceramic top coat. This function prevents the spallation of the ceramic layer during thermal cycling. In addition, the inherent surface roughness of the bond coat enhances the mechanical interlocking and adhesion with the ceramic top coat.

#### 2.2.1. MCrAlY Metallic Bond Coat

MCrAlY (M = Ni, Co, or their combinations) is currently the most widely used bond coat material, typically fabricated via atmospheric plasma spraying (APS) or high-velocity oxygen-fuel (HVOF) spraying. Ni and Co, as matrix elements, determine the fundamental mechanical properties and thermal stability of the coating, while Al and Cr provide oxidation and hot corrosion resistance through the formation of protective Al_2_O_3_ and Cr_2_O_3_ oxide scales, respectively. In addition, reactive elements (REs), such as Y, Hf, and Si, significantly enhance coating durability by purifying impurities, improving oxide scale adhesion, and optimizing the microstructure [54]. However, the optimal concentration of reactive elements is not constant. When the coating thickness increases excessively, an “over-doping” effect may occur due to the excessive reserve of REs. In particular, excessive yttrium can rapidly diffuse toward the interface and form coarse, penetrating Y-rich oxide pegs at the oxide scale/metal interface. These coarse oxides fail to relieve internal stress effectively and instead act as stress concentration sites and diffusion pathways, accelerating oxygen ingress and increasing the oxide growth rate, ultimately deteriorating the high-temperature oxidation resistance of the coating [55]. Furthermore, the incorporation of second-phase particles (e.g., B_4_C, Al_2_O_3_, WC-Co) into the NiCrAlY matrix can significantly improve coating performance, including an increase in hardness of up to 76%, as well as enhanced densification and high-temperature stability [56].

**Figure 5:**
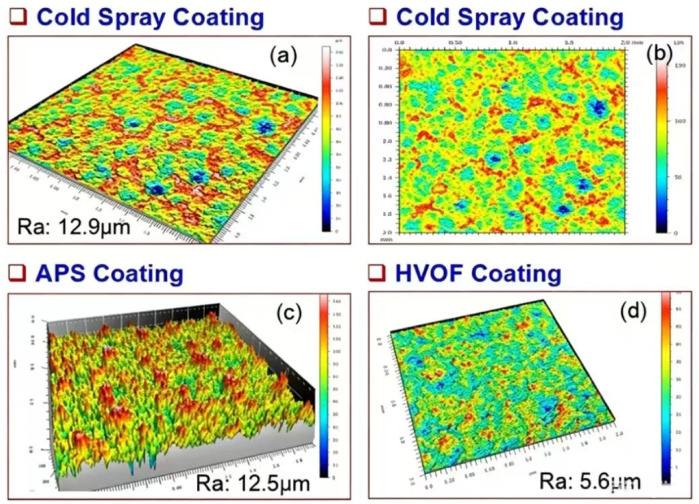
Morphologies of bond coats deposited by cold spraying (CS) (**a**,**b**), air plasma spraying (APS) (**c**), and high-velocity oxygen fuel (HVOF) spraying (**d**) [60].

The high-temperature failure behavior of MCrAlY bond coats in TBC systems is governed by the combined effects of TGO stress evolution, Al_2_O_3_ phase transformation, brittle phase formation, and interfacial sulfur segregation. Studies have shown that the thermal expansion mismatch between the TGO and the bond coat is the primary source of thermal stress. Meanwhile, depletion of Al promotes oxidation of Ni and Co, leading to the formation of brittle spinel phases and degradation of interfacial integrity. Sulfur segregation at elevated temperatures further reduces interfacial bonding strength and promotes void nucleation. To mitigate these degradation mechanisms, various modification strategies have been developed, including vacuum heat treatment, pre-oxidation, surface grain refinement, shot peening, and doping with reactive elements (e.g., Hf, Ta, Ce) or nanoparticles (e.g., CeO_2_). These approaches can effectively enhance interfacial adhesion, suppress excessive TGO growth, and delay the formation of brittle phases [57]. Eriksson et al. demonstrated that increasing the interface roughness between the bond coat and ceramic top coat significantly improves thermal fatigue life. This improvement is attributed to the early deflection of cracks from the interface into the ceramic layer, thereby reducing local stress intensity [58]. Sigaroodi et al. [59] reported that bond coats fabricated by APS exhibit superior corrosion resistance compared with those prepared by HVOF, primarily due to the formation of a thicker Al-rich TGO layer that effectively neutralizes molten salts and delays coating failure. Corporation et al. [60] compared cold-sprayed MCrAlY bond coats with those prepared by APS and HVOF. The results showed that cold-sprayed coatings possess a dense, oxide-free microstructure and a unique honeycomb-like surface morphology (Figure 5), featuring both long-range and short-range roughness. TBC systems incorporating cold-sprayed bond coats exhibited spallation resistance comparable to APS coatings and superior to HVOF coatings. The thickness of MCrAlY bond coats is typically controlled within the range of 100 to 300 μm to ensure sufficient oxidation resistance and stress buffer capability.

#### 2.2.2. Platinum (Pt)-Modified Aluminide

Platinum (Pt)-modified aluminide systems have gradually become the most mature and widely adopted technical route for high-temperature protective bond coats. The incorporation of Pt significantly enhances the adhesion between oxide scales and the substrate. This improvement is primarily attributed to the ability of Pt to suppress the interfacial segregation of detrimental impurities such as sulfur, carbon, and refractory elements, thereby purifying the interface and preventing scale spallation induced by impurity accumulation [61,62]. Meanwhile, Pt effectively modifies the thermodynamic activity and diffusion kinetics of aluminum, promoting the outward flux of Al from the coating. This facilitates the preferential formation of a continuous, dense, and slow-growing α-Al_2_O_3_ thermally grown oxide (TGO) layer during the early stages of oxidation, while suppressing the formation of secondary oxides such as NiO and spinel phases [63]. Sun et al. [64] prepared three types of coatings—NiCrAlYSi, Pt + NiCrAlYSi, and NiCrAlYSi + Pt—via arc ion plating and electroplating. Their results demonstrated that when Pt is distributed in the outer layer (NiCrAlYSi + Pt), the oxidation resistance is significantly enhanced. After isothermal oxidation at 1100 °C for 300 h, this coating exhibited the lowest weight gain (1.37 mg/cm^2^). Pint et al. [65] further reported that Pt diffusion coatings can markedly extend the service life of thermal barrier coatings (TBCs) on N5 and N6 superalloys. These coatings outperform conventional Pt-modified aluminide coatings, and the substrate composition exerts a significant influence on coating performance. Tolpygo et al. [66] vestigated the cyclic oxidation behavior of (Ni,Pt)Al bond coats and revealed that surface rumpling is not primarily governed by oxide scale cracking, thermal expansion mismatch, or growth stress. Instead, it originates from the decomposition of β-(Ni,Pt)Al into γ′-Ni_3_Al due to continuous aluminum depletion. This phase transformation induces local volume contraction, leading to surface deformation. The resulting rumpling promotes local delamination at the TBC/TGO interface, and these delaminated regions progressively accumulate and coalesce during thermal cycling, ultimately forming critical defects that trigger coating spallation.

**Figure 6:**
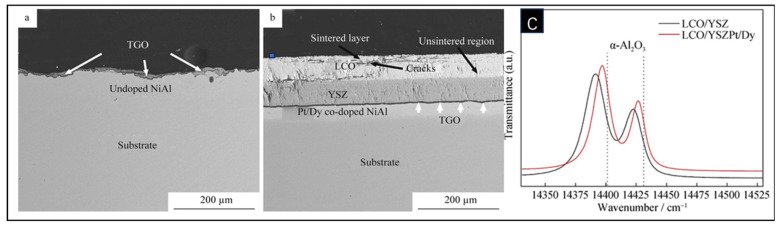
Cross-sectional SEM images of DCL coatings with (**a**) undoped and (**b**) Pt/Dy co-doped NiAl bond coats after 1200 thermal cycles at 1150 °C; (**c**) luminescence spectra of DCL coatings with undoped and Pt/Dy co-doped NiAl bond coats after ~500 thermal cycles [71].

To address the interdiffusion issue between the bond coat and substrate, the introduction of refractory alloying elements has been proposed as an effective strategy. Li et al. [67] successfully fabricated Re-doped β-(Ni,Pt)Al coatings on high-Mo IC21 single-crystal superalloys. Compared with conventional PtAl coatings, the Re-modified coatings exhibited significantly improved spallation resistance and oxide scale continuity after 300 h of oxidation. This enhancement is attributed to the formation of σ-MoRe phases at the coating/substrate interface and grain boundaries, which effectively suppress Mo outward diffusion, prevent the formation of volatile Mo oxides, and improve oxide scale adhesion. In addition to Re, the incorporation of Ru can increase defect formation energy, thereby inhibiting Al diffusion, retarding phase transformation, and suppressing the precipitation of topologically close-packed (TCP) phases. Acting as an effective diffusion barrier, the RuNiAl layer significantly reduces interdiffusion between the coating and substrate, delays the formation of secondary reaction zones (SRZ), and enhances the structural stability and oxidation resistance of the coating [68,69]. Regarding rare-earth modification, Zhang et al. [70] elucidated a dual mechanism by which Dy improves the adhesion of the Al_2_O_3_/NiAl interface. On one hand, Dy forms strong bonds with sulfur impurities, suppressing their interfacial segregation (purification effect). On the other hand, Dy directly participates in interfacial bonding, thereby strengthening interfacial cohesion. Building upon this mechanism, Zhou et al. [71] introduced Pt/Dy co-doped NiAl bond coats into La_2_Ce_2_O_7_/YSZ dual-ceramic-layer TBC systems. The results showed that co-doping more than doubled the thermal cycling lifetime, while effectively suppressing spinel formation in the TGO layer, reducing residual stresses, and inhibiting the precipitation of TCP phases in the substrate (Figure 6).

#### 2.2.3. High-Entropy Alloy Bond Coat

Conventional MCrAlY bond coats suffer from evident performance degradation at temperatures exceeding 1100 °C. In contrast, high-entropy alloys (HEAs), benefiting from the high-entropy effect, sluggish diffusion effect, and severe lattice distortion effect, exhibit superior high-temperature stability, oxidation resistance, and improved thermal expansion compatibility with ceramic top coats.

Studies have demonstrated that AlCoCrFeNi-based HEAs can effectively promote the formation of a dense and continuous α-Al_2_O_3_ thermally grown oxide (TGO) layer while suppressing the formation of deleterious spinel phases. As a result, the high-temperature oxidation resistance and hot corrosion resistance of the bond coat are significantly enhanced. Furthermore, the incorporation of reactive elements such as Y and Hf can further optimize interfacial adhesion and reduce the growth rate of oxide scales [72]. Zhao Xiaofeng’s group [73] reported that Y/Hf-modified NiCoCrAlFe HEAs exhibit an in situ nano-coherent microstructure, leading to an exceptionally low oxidation rate constant of 3.4 × 10^−14^ cm^2^/s at 1100 °C, which is markedly superior to that of conventional NiCoCrAlY alloys. Moreover, this alloy maintains excellent oxide scale adhesion even at ultra-high temperatures up to 1200 °C, accompanied by significantly enhanced hot corrosion resistance. Ossiansson et al. [74] fabricated CrFeCoNi and AlCrFeCoNi HEA bond coats via high-velocity air-fuel (HVAF) spraying. The results showed that AlCrFeCoNi exhibits a lower oxidation rate and higher hardness (approximately 600 HV0.2). However, its thermal cycling lifetime remains inferior to that of commercial MCrAlY coatings containing reactive elements. Although the addition of Al markedly improves oxidation resistance, further incorporation of reactive elements such as Y, Hf, and Si is still required to achieve performance comparable to commercial systems, particularly in terms of oxide scale integrity and interfacial adhesion.

Currently, MCrAlY and Pt-modified aluminide remain the most commonly used bond coat systems in aero-engines, while high-entropy alloy bond coats have attracted widespread attention in recent years due to their excellent high-temperature stability and resistance to interdiffusion. Table 2 summarizes the composition, thickness range, advantages and disadvantages, and common preparation processes of typical TBC bond coat systems.

As shown in Table 2, the conventional MCrAlY bond coat remains the most widely used system in industrial applications due to its good oxidation resistance and mature processing technology. The Pt-modified aluminide offers superior TGO adhesion but comes with higher cost. The high-entropy bond coat exhibits outstanding high-temperature stability and diffusion barrier performance; however, its long-term service reliability still requires further validation.

## 3. Advances in T Environmental Barrier Coatings

With the continuous increase in aero-engine thrust-to-weight ratios, high-temperature components such as thrust chambers and turbine sections have begun to adopt lightweight, high-strength, and high-temperature-resistant ceramic matrix composites (CMCs). However, CMCs are highly susceptible to oxidation in high-temperature, water-vapor-rich environments, reacting with water vapor to form gaseous silicon hydroxide, which leads to continuous material volatilization, thinning, and eventual failure. To address this issue, Environmental Barrier Coatings (EBCs) have emerged as a core technology to ensure the safe service of CMC components. Unlike the Thermal Barrier Coatings (TBCs) described in the previous chapter, which are primarily applied to metallic turbine blades and vanes with “thermal insulation” as their primary function—i.e., when facing ultra-high temperatures of 1400–1600 °C, they achieve a temperature reduction of 100–300 °C on the substrate through extremely low thermal conductivity, ensuring that the metallic substrate remains within a safe operating range below 1000 °C—the primary mission of EBCs is “protection.” EBCs are widely used on CMC components such as combustion chamber liners, nozzles, and turbine guide vanes. They are designed to form a physical and chemical barrier that rigorously prevents high-temperature water vapor from reacting with the CMC substrate to generate gaseous silicon hydroxide, thereby enabling the CMC components to withstand surface temperatures of 1200–1400 °C and achieving significant improvements in engine thrust and efficiency.

**Figure 7:**
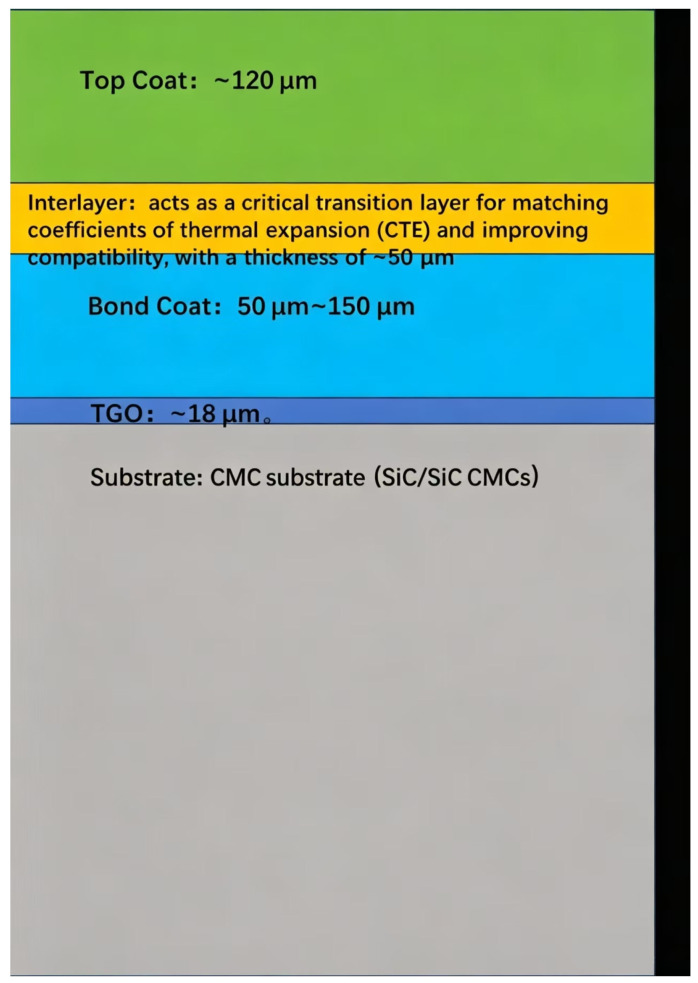
Schematic of the EBC system structure.

A typical EBC system usually consists of a silicon bond coat, an intermediate layer (e.g., mullite), and a silicate top coat, as shown in Figure 7. The silicon bond coat, typically controlled at a thickness of 50–150 μm, primarily serves to provide excellent adhesion and to form a dense SiO_2_ protective layer at high temperatures. In terms of academic evolution, the research focus of EBCs has shifted from early single-layer mullite systems to multi-layer composite systems represented by rare-earth silicates (RE-silicates), pursuing better high-temperature stability and thermal expansion matching. However, current EBCs still face durability challenges such as spallation under extreme thermal gradients during service. Therefore, the following sections of this chapter will provide a detailed review of rare-earth silicates and emerging high-entropy systems, exploring how composition regulation can enhance protective performance and construct a new generation of integrated “thermal/environmental protection” coating systems.

### 3.1. Environmental Barrier Coating Bond Coat

To meet the thermal protection requirements of next-generation ceramic matrix composites (CMCs), the bond coat in environmental barrier coating (EBC) systems serves as a critical interfacial layer connecting the substrate and the top coat. Its performance directly governs the service durability and structural reliability of the entire coating system.

**Figure 8:**
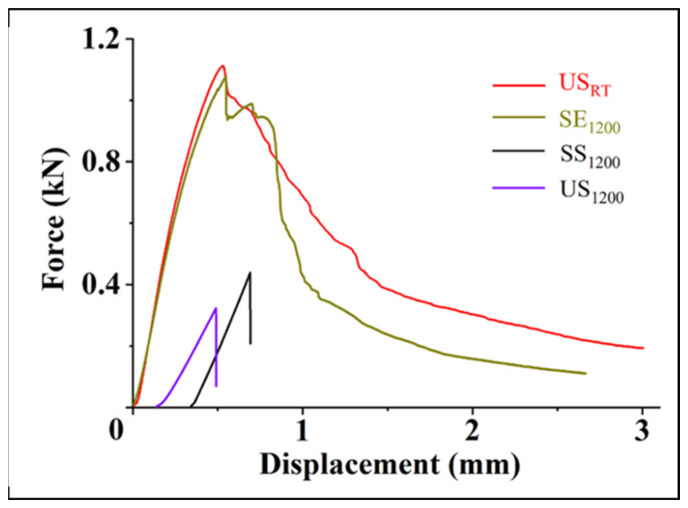
Force–displacement curves of USRT, SE1300, SS1300, and US1300 [80].

Conventional EBC systems predominantly employ pure silicon as the bond coat [75,76,77]. Its primary function is to form a dense SiO_2_ thermally grown oxide (TGO) layer through high-temperature oxidation, thereby providing environmental protection. However, during cooling, the phase transformation of SiO_2_ from β-cristobalite to α-cristobalite is accompanied by significant volume shrinkage, which induces cracking within the TGO layer and subsequently leads to coating spallation. Lee et al. [78] further revealed that at temperatures exceeding 1300 °C, the TGO layer reacts with barium strontium aluminosilicate (BSAS) in the top coat to form low-melting-point glassy phases, which accelerates coating degradation and eventual spallation. To address this issue, Chen et al. [79] demonstrated that doping Al_2_O_3_ into the Si bond coat can effectively suppress the β → α cristobalite phase transformation of SiO_2_, thereby mitigating volume shrinkage and preventing crack initiation. To further alleviate thermal mismatch and enhance fracture resistance, Hu et al. designed a four-layer EBC architecture consisting of Si/Si-Yb_2_SiO_5_/Yb_2_SiO_5_/LaMgAl_11_O_19_, which was deposited onto SiC/SiC composites via atmospheric plasma spraying (APS). After 500 h of isothermal oxidation at 1300 °C, the coated specimens retained 97.25% of their flexural strength (Figure 8) and exhibited pseudo-plastic fracture behavior. Notably, the reaction between Yb_2_SiO_5_ and thermally grown SiO_2_ led to the in situ formation of a dense Yb_2_Si_2_O_7_ layer, which effectively controlled TGO thickness and suppressed crack propagation [80]. Similarly, Li et al. [81] developed a three-layer EBC system composed of Si/Si-Yb_2_SiO_5_/Yb_2_SiO_5_-SiC. In this design, the Si-Yb_2_SiO_5_ interlayer and the Yb_2_SiO_5_-SiC top layer synergistically enable crack self-healing and thermal stress regulation. The coating exhibited no observable spallation after 800 thermal shock cycles at 1300 °C, demonstrating outstanding oxidation resistance and thermal shock durability.

As pure Si bond coats are intrinsically limited by the melting point of silicon (1414 °C) [82], the development of composite bond coats incorporating high-melting-point ceramic phases has become an inevitable trend. Deijkers et al. determined the kinetic parameters of the reaction between β-cristobalite and monoclinic HfO_2_ for the formation of hafnium silicate. Their results revealed that the reaction proceeds at a sufficiently high rate, thereby providing both experimental evidence and kinetic support for employing HfO_2_ as a reactive component in Si-based EBC bond coats [83]. Harder et al. [82] fabricated Si–HfO_2_ composite bond coats using plasma spray–physical vapor deposition (PS-PVD). In the as-deposited state, Si existed in a metallic form with dispersed HfO_2_ particles, and HfSiO_4_ formed locally in certain regions. However, the Si–HfO_2_ bond coat exhibited limited oxidation protection for SiC substrates, with a TGO growth rate comparable to that of uncoated SiC. This result suggests that further optimization of Si content and its spatial distribution is required to enhance coating performance. Similarly, Bakan et al. [84] prepared Si–HfO_2_ coatings via atmospheric plasma spraying (APS). Their findings indicated that incomplete formation of HfSiO_4_ failed to effectively consume SiO_2_, leading to its accumulation and subsequent phase-transformation-induced cracking. Moreover, the formation of HfSi_2_ was identified as a critical factor contributing to coating degradation. Liu et al. [85] successfully fabricated dense and crack-free HfO_2_–Si bond coats with a porosity below 5% through precise control of APS parameters. However, as the HfO_2_ content increased, incomplete melting of HfO_2_ resulted in a gradual reduction in coating thickness (from 55 μm to 31 μm). In addition, pronounced phase separation was observed within the coatings, highlighting the intrinsic limitations of conventional mechanical mixing approaches.

Unlike conventional TBC systems, environmental barrier coatings (EBCs) are required not only to reduce the surface temperature of the substrate but also to protect Si-based composites in high-temperature water vapor environments. Table 3 summarizes the composition, thickness range, and main functions of typical EBC bond coat/interlayer systems. As shown in Table 3, current EBCs mostly adopt a multilayer synergistic protection structure. The Si bond coat provides underlying protection, while the rare-earth silicate topcoat determines the high-temperature water vapor stability. With increasing service temperatures, multilayer composite structures and high-entropy rare-earth silicate systems are gradually becoming research focuses.

### 3.2. Environmental Barrier Coating Top Coats

In aero-engine combustion environments containing high-temperature water vapor, silicon-based ceramics such as SiC undergo severe degradation. The dense silica (SiO_2_) scale that provides protection under dry air conditions becomes unstable in water vapor, where it reacts to form volatile silicon hydroxides. This leads to continuous matrix recession of silicon-based ceramics. Such linear recession rapidly deteriorates the mechanical integrity of critical components, including turbine blades and combustion chamber liners. To address this issue, coating design has evolved beyond simple resistance to corrosive species toward the development of environmental barrier coatings (EBCs), which aim to effectively isolate the substrate from water vapor exposure, thereby mitigating vapor-induced degradation [3]. From a historical perspective, first-generation EBCs were primarily based on simple oxides, with mullite as a representative material. Although mullite exhibits superior thermodynamic stability compared with SiC, it inherently contains SiO_2_. Under high-velocity water vapor environments, selective volatilization of silicon occurs at the coating surface, resulting in increased porosity. This porous structure facilitates water vapor penetration and accelerates substrate degradation. Therefore, single-layer mullite coatings can only delay, rather than fundamentally prevent, environmental attack [3]. Second-generation EBCs introduced barium strontium aluminosilicate (BSAS) systems to enhance water vapor resistance [86]. While these coatings demonstrated promising performance in early laboratory tests, their long-term stability remains limited. Under extreme water vapor conditions, both barium and silicon in BSAS undergo chemical reactions, forming volatile hydroxides such as Ba(OH)_2_ and Si(OH)_4_. This simultaneous depletion of multiple constituents leads to coating thinning and deviation from the original stoichiometry. Furthermore, severe Ba depletion induces phase transformation accompanied by substantial volume changes, which promote crack initiation [87]. The combined effects of chemical volatilization and mechanical spallation result in insufficient durability for long-term service requirements (e.g., 30,000 h) in real engine environments [88]. To achieve higher operating temperatures and improved durability, third-generation rare-earth silicates (RE_2_SiO_5_ and RE_2_Si_2_O_7_) have been developed. Lee et al. [89] demonstrated that, compared with BSAS, these materials exhibit significantly lower volatilization rates, effectively mitigating the rapid recession of SiC-based substrates under high-temperature water vapor conditions. For Si_3_N_4_ substrates, rare-earth silicate coatings not only block external water vapor but also suppress undesirable chemical interactions with internal sintering additives.

**Figure 9:**
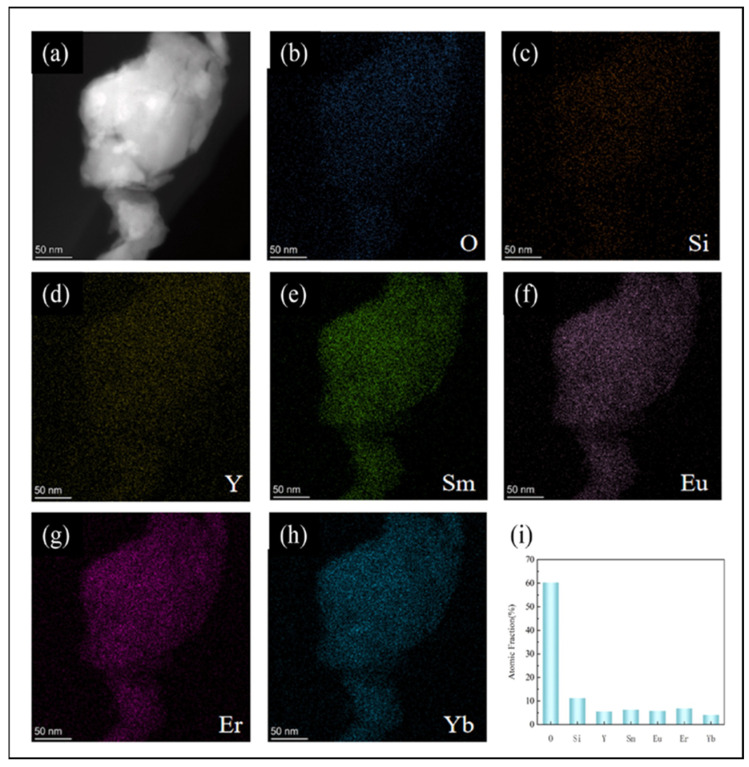
(**a**) TEM image of (Y_0.2_Sm_0.2_Eu_0.2_Er_0.2_Yb_0.2_)_2_SiO_5_; (**b**–**h**) corresponding EDS elemental mappings of O, Si, Y, Sm, Eu, Er, and Yb, respectively; (**i**) atomic fractions of the as-prepared specimen [96].

With the increasing demand for higher thrust-to-weight ratios and thermal efficiency, recent research has shifted toward high-entropy ceramic systems [90]. Turcer et al. [91] reported that high-entropy rare-earth pyrosilicates, designed through compositional disorder, exhibit ultralow thermal conductivity. The highly distorted lattice significantly suppresses phonon transport, approaching the theoretical minimum of amorphous solids. Consequently, these materials combine the thermal stability of crystalline ceramics with the low thermal conductivity of glass, making them promising candidates for integrated thermal/environmental barrier coatings (TEBCs) [92]. Sun et al. [93] further developed a six-component equiatomic high-entropy system (Gd, Tb, Dy, Tm, Yb, Lu). Owing to the large ionic radius mismatch and high configurational entropy, the system is stabilized into a single-phase γ structure. Experimental results show that the coating maintains a dense single-phase γ structure even after prolonged annealing at temperatures above 1500 °C, without any detectable secondary phases or γ-to-δ phase transformation. This effectively eliminates phase-transformation-induced stresses during service, confirming the exceptional thermal stability of such high-entropy systems. In parallel, Dong [94] and Chen et al. [95] demonstrated that high-entropy materials ssynthesized via sol–gel methods exhibit superior resistance to water vapor volatilization compared with conventional single-component ceramics. Furthermore, Liu et al. [96] developed a green molten-salt synthesis route using NaCl–KCl as the reaction medium. The molten salt provides a low-viscosity liquid environment, significantly enhancing the diffusion rates of rare-earth ions and silicon precursors. This enables a statistically uniform distribution of elements at lattice sites. The resulting powders exhibit high phase purity without residual unreacted oxides (Figure 9), offering an efficient and low-energy pathway for the synthesis of high-purity high-entropy rare-earth silicates.

In the design of advanced multilayer thermal/environmental barrier coatings (TEBCs), achieving a balance among ultra-high temperature resistance, severe corrosion tolerance, and thermal shock durability remains a critical challenge in materials science. To address these demands, a variety of fabrication strategies have been developed. For example, Ramasamy et al. [97] fabricated mullite/gadolinium silicate multilayer EBCs on SiC and Si_3_N_4_ substrates via slurry dip-coating, systematically optimizing slurry chemistry (alcohol-based and sol-based systems) and sintering conditions. In parallel, plasma spray–physical vapor deposition (PS-PVD) [98] has demonstrated unique capability in producing either columnar or highly dense microstructures, both of which significantly enhance coating integrity under extreme service conditions. Beyond structural design, the functionality of multilayer systems relies critically on compositional engineering. The so-called “cocktail effect” of multicomponent rare-earth elements [99,100] enables precise tuning of the coefficient of thermal expansion (CTE), thereby mitigating thermal mismatch stresses between the coating and SiC/SiC substrates at the microscale. This synergistic regulation is essential for suppressing crack initiation and preventing spallation during long-term thermal cycling.

**Figure 10:**
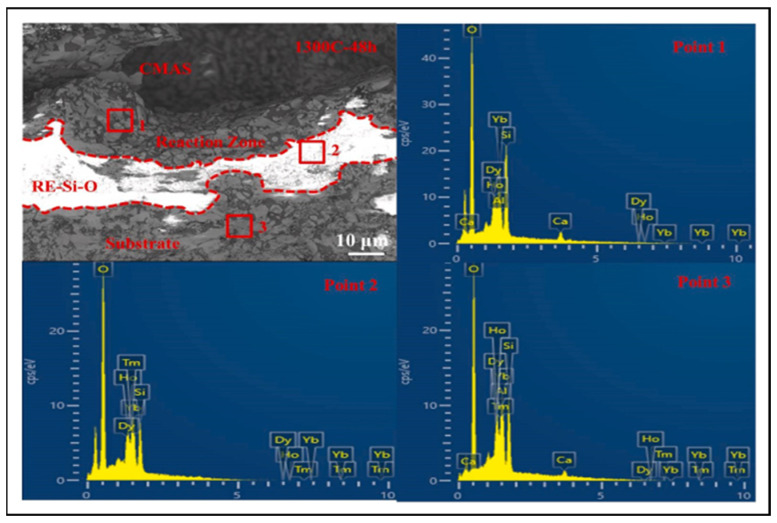
Cross-sectional morphology of CMAS interaction with (4RE_1_/_4_)_2_Si_2_O_7_ at 1300 °C for 48 h, along with corresponding EDS analysis [103].

In addition to thermal and mechanical challenges, TEBCs must withstand calcium–magnesium–aluminosilicate (CMAS) corrosion originating from ingested environmental particulates. Above ~1200 °C, CMAS melts and infiltrates coating porosity, leading to coupled failure driven by chemical dissolution and stress accumulation. Recent studies reveal that high-entropy rare-earth monosilicates and pyrosilicates exhibit a distinctive “active defense” mechanism against CMAS attack. Specifically, rapid in situ reactions between the coating and molten CMAS lead to the formation of dense, continuous reaction layers, typically with apatite- or garnet-type structures [101,102]. These high-melting-point phases effectively seal diffusion pathways and suppress further penetration of molten salts. Abrar et al. [103] ssynthesized an equimolar high-entropy pyrosilicate, (Dy_1_/_4_Ho_1_/_4_Tm_1_/_4_Yb_1_/_4_)_2_Si_2_O_7_, exhibiting a stable single-phase β structure. After 48 h of CMAS exposure at 1300 °C, a dense apatite reaction layer and a Ca-depleted recrystallized rare-earth silicate layer were formed on the surface, effectively inhibiting CMAS infiltration (Figure 10). The study further elucidated the functional role of cation size distribution: large-radius ions (Dy, Ho) preferentially participate in apatite formation; small-radius ions (Yb) contribute to interfacial stabilization and protection; and intermediate-radius ions (Tm) maintain the structural stability of the pyrosilicate matrix. These findings highlight the effectiveness of high-entropy design in enhancing CMAS resistance.

Looking forward, the research paradigm of TEBCs is undergoing a fundamental transition. Rather than relying on the simple accumulation of individual properties such as thermal insulation or oxidation resistance, future efforts should emphasize the property tailorability enabled by high-entropy effects. By precisely controlling the occupation of rare-earth elements with different ionic radii and electronegativities at the atomic scale, it becomes possible to simultaneously optimize fracture toughness, thermal transport behavior, and chemical stability, while maintaining single-phase stability above 1500 °C. This design philosophy represents a shift from conventional passive barrier coatings toward adaptive, chemically responsive systems, capable of initiating beneficial reactions and accommodating damage evolution during service. Such an “active defense” strategy provides a promising pathway for next-generation TEBCs operating under extreme environments.

To further compare the performance of typical environmental barrier coating (EBC) topcoat materials in high-temperature water vapor and CMAS corrosive environments, Table 4 summarizes the thermal conductivity, CTE, thickness, CMAS resistance, and engineering application evaluation of commonly used EBC topcoat materials. As can be seen from Table 4, rare-earth silicate systems exhibit excellent high-temperature water vapor stability and CMAS resistance, representing the current core direction of EBC research and engineering applications; high-entropy rare-earth silicates and materials such as Hf_6_Ta_2_O_17_ show higher potential for long-term thermal cycling and extreme environments, although their complex preparation and cost still need to be considered. This information provides fundamental data for the analysis of high-temperature degradation mechanisms of EBCs in the next chapter.

## 4. Degradation Mechanisms

The preceding chapter discussed in detail the compositional evolution and structural optimization of ceramic topcoat materials (TBCs and EBCs) as well as metallic bond coats. However, under the extreme service conditions encountered in real aero-engine operation, the performance of coating systems is not independent; their long-term stability is profoundly influenced by the coupled effects of high-temperature oxidation, environmental contaminant attack, and thermomechanical loading. As underscored by engineering failure analysis [104], while catastrophic in-service failures are rare due to stringent inspection regimes, the rejection rate of turbine components during overhaul due to incipient failure symptoms remains significant. This highlights a critical gap between idealized laboratory performance and the multi-axial degradation experienced in real-world service. With further increases in turbine inlet temperatures, the degradation mechanisms faced by coatings have evolved from simple thermal mismatch stresses to complex chemo-mechanical interactions.

From an evolutionary perspective, while the bond coat provides adhesion, the thermally grown oxide (TGO) layer formed on its surface is the key factor determining the service life of the system. However, environmental deposits such as calcium-magnesium-aluminosilicate (CMAS), water vapor, and molten salts from fuel impurities can penetrate through the pores or cracks of the ceramic layer and undergo intense physiochemical reactions with the coating. These environmental attacks trigger a synergistic degradation chain: the thermochemical destabilization of the ceramic topcoat accelerates the depletion of the bond coat’s protective capacity, eventually leading to structural compromise—such as creep or fatigue—of the underlying superalloy or CMC substrate [104]. Therefore, an in-depth understanding of these degradation mechanisms is crucial for developing next-generation coating systems with “active protection” capabilities. This chapter will focus on analyzing the effects of CMAS attack, steam erosion, and molten salt corrosion on the structural integrity of coatings.

### 4.1. CMAS Corrosion Mechanism

During service, aero-engines and gas turbines inevitably ingest environmental particulates, such as sand, volcanic ash, and runway debris. At elevated temperatures (typically above 1200 °C), these particles melt to form calcium–magnesium–aluminosilicate (CMAS) glassy deposits [7,105]. Owing to their high fluidity and strong chemical reactivity, CMAS melts readily adhere to hot-section components and are widely recognized as one of the primary causes of premature failure in thermal barrier coatings (TBCs) and environmental barrier coatings (EBCs) [106].

**Figure 11:**
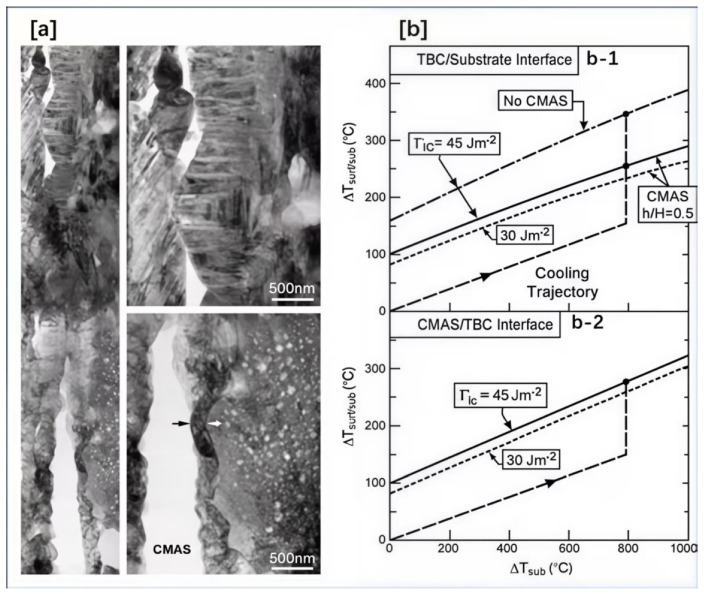
Comparative schematics of CMAS infiltration progression and failure modes in TBCs with different microstructures: (**a**) capillary infiltration and reactive sintering in a columnar EB-PVD coating [106]; (**b-1**,**b-2**) progression of infiltration, cracking, and delamination within a lamellar APS coating system [110] (**b**).

In conventional TBC systems, 7–8 wt.% yttria-stabilized zirconia (YSZ) is particularly susceptible to CMAS attack due to its porous or columnar microstructure [107,108]. Krämer et al. demonstrated that molten CMAS rapidly infiltrates YSZ through capillary forces, filling pores and interlamellar gaps. This process induces the selective dissolution of the Y_2_O_3_ stabilizer and triggers the deleterious phase transformation from metastable tetragonal (t′) to monoclinic (m) zirconia [106]. The combined effects of physical infiltration and chemical degradation not only significantly increase the thermal conductivity of the coating [109], but also severely reduce its strain tolerance. Consequently, thermal mismatch during cooling leads to the formation of vertical channel cracks and interfacial spallation cracks [110,111,112]. The distinct architectural differences between these coatings lead to divergent failure trajectories, as schematically illustrated in Figure 11. In Electron Beam-Physical Vapor Deposition (EB-PVD) coatings (Figure 11a), the molten CMAS rapidly fills the prominent intercolumnar gaps via capillary action. This process triggers a dissolution-reprecipitation mechanism that fuses the columns into a monolithic, brittle layer, causing a catastrophic loss of the system’s inherent strain tolerance [106]. Conversely, in Atmospheric Plasma Sprayed (APS) coatings (Figure 11b), the infiltration follows a more tortuous path through a network of interconnected splat boundaries and globular pores. The accumulation of CMAS-induced stresses in these systems typically leads to horizontal crack propagation and eventual large-scale delamination within the penetrated zone [110].”Zhou et al. further investigated nanostructured YSZ coatings prepared by atmospheric plasma spraying (APS). At low CMAS contents, corrosion preferentially occurs along grain boundaries, where Ca^2+^ and Mg^2+^ diffuse into the ZrO_2_ lattice, forming cubic zirconia (c-ZrO_2_). As the CMAS content increases, the degradation mechanism transitions to a dissolution–reprecipitation process: YSZ is progressively dissolved by molten CMAS, accompanied by outward diffusion of Y^3+^ and resultant yttrium depletion. Upon cooling, the t′ → m transformation occurs, and the associated volume expansion exacerbates internal stress accumulation. Moreover, nanostructured YSZ, characterized by a Y-rich surface and high specific surface area, is more prone to such reactions under severe CMAS exposure, leading to increased formation of m-ZrO_2_. Its higher porosity further accelerates CMAS infiltration [113].

To mitigate CMAS-induced degradation, advanced rare-earth zirconate coatings, such as Gd_2_Zr_2_O_7_, have been developed. Their protection mechanism relies on rapid chemical reactions with CMAS melts, inducing the precipitation of high-melting-point apatite phases, which produce a self-sealing effect that effectively blocks further melt penetration [114]. Zou Lanxin et al. [115] fabricated nanostructured YSZ (NYSZ) and multi-component rare-earth oxide-modified zirconia (MSZ) coatings via APS. Compared with NYSZ, MSZ coatings—characterized by lower specific surface area, absence of nanostructure, and superior phase stability (dominated by the cubic phase)—exhibit significantly reduced monoclinic phase formation after CMAS exposure, resulting in enhanced resistance to CMAS wetting and corrosion.

**Figure 12:**
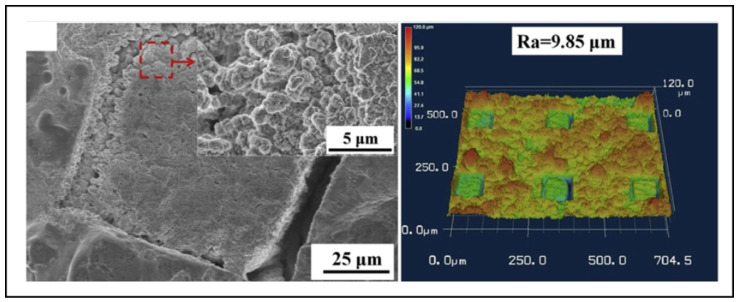
Surface morphologies of YSZ coatings with different structures: femtosecond laser-ablated coating [115].

Beyond compositional optimization, recent studies have emphasized microstructural design to suppress CMAS attack. Kang et al. [116] constructed periodic square-pit microstructures on YSZ surfaces using femtosecond laser processing, featuring internal micro-rod morphologies (Figure 12). Despite a relatively low initial contact angle, the structure effectively inhibits CMAS spreading and infiltration by pinning the triple-phase contact line and establishing a Cassie-state wetting regime between micro-rods. As a result, a higher equilibrium contact angle is achieved, leading to improved CMAS resistance compared with untreated or polished coatings. Yan et al. [117] employed millisecond laser remelting (laser glazing) to generate a dense columnar remelted layer on APS-YSZ coatings. The remelted layer exhibits a smooth surface accompanied by a network of vertical cracks. After 10 h of CMAS exposure at 1250 °C, it maintains excellent phase stability without noticeable t′ → m transformation. However, vertical cracks and intercolumnar gaps act as fast diffusion pathways for CMAS, leading to severe dissolution–reprecipitation damage in the underlying coating. In extreme cases, this results in buckling or spallation of the remelted layer. These findings indicate that although laser glazing enhances surface resistance, its inherent open-channel structure requires further optimization. Additionally, pore structure regulation via sol–gel impregnation has been demonstrated to effectively improve anti-wetting behavior and physical barrier performance [118].

Compared with TBCs, the failure mechanisms of environmental barrier coatings (EBCs), particularly rare-earth silicates such as Yb_2_Si_2_O_7_ and Lu_2_Si_2_O_7_, exhibit a stronger thermochemical dependence under CMAS corrosion conditions [119]. Turcer et al. systematically compared the interactions between CMAS glass and yttrium-containing versus yttrium-free EBC ceramics at 1500 °C, revealing that yttrium-free β-Yb_2_Si_2_O_7_ undergoes negligible reactive crystallization. Nevertheless, in both systems, CMAS melt gradually infiltrates along grain boundaries, generating a through-thickness expansion gradient that induces a distinct high-temperature blistering-type cracking failure. Based on this mechanism, an effective mitigation strategy has been proposed: introducing 1 vol% CMAS glass into β-Yb_2_Si_2_O_7_ powders prior to sintering to pre-distribute CMAS phases along grain boundaries. This approach promotes more uniform CMAS infiltration, thereby suppressing the formation of expansion gradients and significantly mitigating blistering-induced cracking [120]. In contrast, monosilicate systems typically suffer more severe CMAS-induced degradation. The coating matrix tends to dissolve into the molten CMAS, forming complex silicate glass layers and precipitating calcium-rich silicate phases. Although these reaction products may partially retard further corrosion, the associated volume changes and localized stress concentrations inevitably lead to coating cracking [115]. A fundamental distinction between EBCs and TBCs lies in their protected substrates, which are predominantly SiC/SiC ceramic matrix composites (CMCs). These substrates are highly sensitive to oxygen, water vapor, and CMAS attack [7,121]. Once CMAS-induced damage compromises the densification and integrity of EBCs, molten salts and corrosive species can directly penetrate and attack the CMC substrate, resulting in rapid oxidation, decarburization, and irreversible structural degradation.

In recent years, multi-component high-entropy design strategies have demonstrated great potential in enhancing CMAS resistance. Sun et al. investigated the interaction between CMAS melts and multi-component β-(Er_0.25_Tm_0.25_Yb_0.25_Lu_0.25_)_2_Si_2_O_7_ coatings at 1500 °C. The results indicate that this high-entropy system exhibits superior CMAS resistance compared with single-component rare-earth disilicates. Notably, the typical blister-like cracking observed in conventional systems is absent, and residual CMAS glass remains in the reaction zone, suggesting a reduced dissolution rate and enhanced grain boundary stability [115]. Overall, current research is transitioning from the conventional paradigm of single-property optimization toward multi-scale coupled design. Li et al. systematically evaluated four A_6_B_2_O_17_ (A = Zr/Hf, B = Nb/Ta) ceramics and identified Hf_6_Ta_2_O_17_ as exhibiting the most superior CMAS resistance. The B-site elements (Nb, Ta) govern the reverse dissolution process through optical basicity; specifically, the high optical basicity of Nb promotes the formation of a dense reaction layer, which inhibits Si diffusion and suppresses HfSiO_4_ formation. Meanwhile, the A-site elements (Zr, Hf) regulate the stability of corrosion products through AO_2_ formation. The relatively low phase transition temperature of ZrO_2_ leads to crack initiation and spallation, thereby accelerating degradation. In contrast, Hf_6_Ta_2_O_17_ forms a dense composite layer consisting of CaTa_2_O_6_ and HfSiO_4_ during CMAS exposure, effectively sealing surface pores and blocking further melt infiltration, thus exhibiting outstanding corrosion resistance. Looking forward, future strategies should integrate the intrinsic chemical inertness of advanced ceramics such as A_6_B_2_O_17_ [122] with advanced coating technologies, including nanostructured coating design [113,115] and surface modification techniques. Such an integrated approach enables the development of a comprehensive protection system that combines physical barrier effects, chemical passivation, and mechanical tolerance, thereby addressing the increasingly severe high-temperature corrosion challenges in next-generation aero-engines. This integration is vital because CMAS infiltration not only degrades the coating’s thermal properties but also alters the surface stress state, potentially serving as a nucleation site for thermomechanical fatigue cracks [104].

### 4.2. Water–Oxygen Corrosion Mechanism

In the high-temperature combustion environment of aero-engines, water vapor is not only a major combustion product but also a critical factor governing the degradation of ceramic matrix composites (CMCs) and their protective coatings. The fundamental mechanism of water–oxygen corrosion arises from the thermochemical reaction between the protective SiO_2_ scale formed on silicon-based materials and high-temperature, high-pressure water vapor, producing volatile silicon hydroxide species (e.g., Si(OH)_4_). This reaction leads to continuous material recession and ultimately the loss of structural integrity [121,123]. Moreover, the volatilization rate increases markedly with gas pressure and flow velocity, rendering the application of environmental barrier coatings (EBCs) indispensable [121]. Regarding the water–oxygen corrosion resistance of EBC systems, Al Nasiri et al. [124] conducted a comparative study on five rare-earth monosilicates (RE_2_SiO_5_, RE = Y, Gd, Er, Yb, Lu) at 1350 °C. Although all compositions exhibit excellent chemical stability, their performance strongly depends on the ionic radius of the rare-earth cations. With the continued advancement of materials design, high-entropy strategies have emerged as an effective route to enhance both thermophysical properties and corrosion resistance. Zhang Tao et al. [112] compared high-entropy pyrosilicates with single-component Yb_2_Si_2_O_7_ and demonstrated that high-entropy design not only improves lattice stability but, more importantly, alters the pore evolution behavior within the coating. Specifically, water vapor transport at elevated temperatures is governed by the combined effects of molecular diffusion in macropores and Knudsen diffusion in micropores. High-entropy compositions effectively suppress pore coarsening and interconnection, thereby reducing the inward permeation of corrosive species. Furthermore, microstructural engineering plays a crucial role in controlling corrosion kinetics. Wang et al. [125] fabricated Yb_2_SiO_5_ coatings with a characteristic columnar structure via plasma spray–physical vapor deposition (PS-PVD). The unique microstructural features introduced by this technique significantly influence the kinetics of water–oxygen corrosion. It is worth noting that water vapor also exerts a non-negligible influence in thermal barrier coating (TBC) systems. Although TBCs are primarily designed for thermal insulation, water vapor can significantly accelerate the oxidation kinetics of metallic bond coats (BCs) [126]. Cao et al. [127] employed a modified oxidation kinetic model to investigate the corrosion behavior of SiC/SiC composites under different atmospheric conditions. Their results indicate that water–oxygen environments alter the growth behavior of thermally grown oxides (TGO), promoting the formation of porous structures and microcracks. To address this issue, Liang Ruihui et al. [128] proposed Yb_2_O_3_ doping modification in silicon-based bond coats. The incorporation of an appropriate amount of rare-earth oxide effectively mitigates coating cracking induced by abnormal TGO growth and enhances the durability of the coating system at 1350 °C. It is important to distinguish that while water-oxygen attack in EBCs primarily leads to the recession of the ceramic layer itself through volatile Si(OH)_4_, in TBC systems, it acts as an accelerant for the sub-scale oxidation of the metallic bond coat, emphasizing the need for different hermetic sealing strategies for these two systems.

### 4.3. Other Corrosion Mechanisms and Synergistic Effects

In addition to CMAS and water–oxygen corrosion, molten salt corrosion originating from combustion gases, along with the synergistic interaction of multiple corrosive media, creates an increasingly complex service environment. Statistical data from engine maintenance reveals that hot corrosion, particularly induced by sodium-rich intake contaminants in marine environments, is a primary driver for premature component rejection [104]. Wan Xiafeng et al. [126] systematically elucidated the hot corrosion mechanisms induced by Na_2_SO_4_ and V_2_O_5_ on thermal barrier coatings (TBCs). In these scenarios, molten salts function as aggressive electrolytes, initiating electrochemical reactions that deplete the aluminum reservoir in the bond coat and trigger the premature “peeling” of the ceramic topcoat [104,126].

The severity of this degradation is closely tied to operational maintenance; for instance, “compressor washing” is employed to reduce the salt load on compressor blades, but if not performed thoroughly, residual deposits can migrate to the hot section, intensifying the corrosion load on turbine coatings [104]. During hot corrosion, molten salts not only destabilize the YSZ phase and induce phase transformations, but also involve complex electrochemical reactions that accelerate premature spallation of the metallic bond coat. Mohan et al. [129] further demonstrated that the coexistence of fuel-derived impurities and CMAS significantly aggravates coating degradation. This synergistic attack can be effectively mitigated by introducing dense barrier layers, such as Al_2_O_3_ or MgO, onto the coating surface. In recent years, research has increasingly focused on synergistic degradation under multi-field coupling conditions. Chen Zhilin [7] and Ashofteh [121] both emphasized the distinctive characteristics of CMAS–water vapor coupled environments. The presence of water vapor may alter melt viscosity and reduce interfacial energy, thereby facilitating deeper CMAS infiltration into coating interiors. Sun et al. [115] reported that, in quaternary rare-earth pyrosilicates, the material degradation rate under the combined action of high-temperature water vapor and molten CMAS is significantly higher than that under single-corrosion conditions. This synergistic effect manifests not only in accelerated chemical reaction kinetics but also in the superposition of physical damage. To better simulate realistic service conditions, Drexler [130] and Steinke [131] independently proposed thermal cycling tests incorporating temperature gradients. Their results indicate that, under the combined influence of CMAS attack and thermal gradient stress, coating failure evolves from localized thermochemical erosion to catastrophic bulk spallation driven by severe loss of strain tolerance. Overall, these studies highlight that future protective coating design must move beyond the development of single-phase materials. Whether through optimizing pore transport behavior in EBCs via high-entropy design [132] or suppressing CMAS infiltration through multi-component compositional engineering [7,115], the underlying principle is to enhance phase stability and environmental tolerance by increasing configurational entropy. Future breakthroughs are expected to focus on intelligent, self-healing coating systems capable of dynamically adapting to the coupled “water–oxygen–molten salt–stress” environment, as well as on establishing more accurate lifetime prediction models that closely reflect real service conditions.

### 4.4. Summary of Degradation and Future Outlook

The transition from single-factor laboratory studies to the analysis of multi-field coupling represents the current frontier in coating research. As synthesized from both mechanistic studies and engineering failure reports [104,115], the failure of TBCs/EBCs is rarely the result of a standalone process but rather a synergistic progression of thermochemical erosion, environmental oxidation, and thermomechanical fatigue. Future breakthroughs must move beyond developing chemically inert phases to designing “intelligent” coatings capable of dynamic self-healing and stress-buffering. Furthermore, the development of lifetime prediction models must integrate these real-world engineering variables—such as salt ingestion rates and maintenance cycles—to provide a robust framework for the safe operation of next-generation aero-engines.

## 5. Conclusions and Outlook

This work systematically reviews the evolution of material systems, structural design strategies, and failure mechanisms of thermal barrier coatings (TBCs) and environmental barrier coatings (EBCs) under complex service environments, thereby revealing the development paradigm of next-generation high-temperature protective coatings for aero-engines. Although conventional yttria-stabilized zirconia (YSZ) remains the most widely used top-coat material, its susceptibility to phase transformation and sintering above 1200 °C significantly limits its application in advanced turbine components [112]. In contrast, emerging ceramic systems based on multi-component rare-earth oxides, pyrochlore-type A_2_B_2_O_7_ structures, and high-entropy oxides exhibit lower thermal conductivity, superior phase stability, and enhanced resistance to CMAS corrosion, making them promising candidates for next-generation TBC systems [132]. Meanwhile, bond-coat systems have evolved from conventional MCrAlY alloys toward Pt-modified aluminides and high-entropy alloy bond coats with sluggish diffusion characteristics, which effectively improve oxidation resistance and microstructural stability at elevated temperatures [73]. For EBC systems applied to ceramic matrix composites (CMCs), increasing attention has been devoted to simultaneously achieving chemical stability, thermal expansion compatibility, and water-vapor corrosion resistance [133]. From the perspective of degradation mechanisms, thermo–mechanical–chemical coupling-induced interfacial spallation and crack propagation remain the dominant factors governing coating failure during long-term service.

Looking forward, the development of advanced protective coatings is expected to gradually shift from conventional single-function thermal insulation toward integrated “thermal–environmental” protection systems capable of simultaneously resisting heat flux, oxidation, water vapor, CMAS corrosion, and thermo-mechanical fatigue [134]. To further clarify future research directions, a staged development roadmap can be proposed from short-term, mid-term, and long-term perspectives.

In the short term (1–3 years), research is expected to focus on optimizing existing double-ceramic-layer (DCL) architectures to balance the excellent CMAS resistance of Gd_2_Zr_2_O_7_-based ceramics with the superior fracture toughness and thermal cycling durability of YSZ layers. In particular, improving interface design, residual stress regulation, and thermal expansion compatibility between pyrochlore top coats and YSZ interlayers will be critical for extending coating lifetime under realistic thermal-gradient conditions [132]. Meanwhile, for EBC systems, further optimization of multilayer rare-earth silicate architectures and interfacial reliability under water-vapor environments will remain a practical engineering priority [133].

In the mid term (5–10 years), high-entropy oxides and high-entropy rare-earth silicates are expected to become key research directions for both TBC and EBC applications. Benefiting from the “entropy stabilization effect,” these multi-component ceramic systems may enable integrated thermodynamic design strategies combining ultralow thermal conductivity, high phase stability, superior CMAS resistance, and enhanced environmental durability within a single material framework [132,135]. In addition, synergistic approaches involving compositional complexity, defect engineering, and multi-scale phonon scattering are expected to provide new pathways for overcoming the traditional trade-off between thermal insulation capability and fracture toughness.

In the long term (>10 years), the development of intelligent lifetime prediction and health-monitoring technologies will become increasingly important for aero-engine coating systems. Future studies are expected to integrate artificial intelligence, Integrated Computational Materials Engineering (ICME), and digital twin technologies to establish data-driven lifetime prediction models capable of simulating coating evolution under realistic thermo-mechanical-environmental coupling conditions [135]. Furthermore, combining these approaches with physics-informed crack evolution simulations and interfacial degradation analyses associated with thermally grown oxide (TGO) evolution [30] may enable the transition from conventional “post-failure analysis” toward proactive early-warning and real-time reliability assessment of coating systems. Ultimately, achieving engineering-scale implementation of intelligent coating architectures on complex hollow turbine blades and CMC hot-section components will be essential for ensuring the long-term durability and reliability of next-generation aero-engines.

## Figures and Tables

**Table 1:** Physical Properties and CMAS Resistance of Typical TBC Top-Coat Materials [10,11,12,13,14,15,16,17,18,19,20,21,22,23,24,25,26,27,28,29,30,31,32,33,34,35,36,37,38,39,40,41,42,43,44,45,46,47,48,49,50,51,52].

Material	Thermal Conductivity (W·m^−1^·K^−1^)	CTE (10^−6^ K^−1^)	Typical Thickness (μm)	Main Advantages & Limitations	CMAS Resistance	Engineering Evaluation
7YSZ	~2.3	10.5–11.5	200–300	Good toughness, mature technology; prone to sintering at high T	Low	Widely used
4.5YSZ	~3.8	10.5–11.5	200–300	Moderate stability	Low–Medium	Medium
3.7Sc3.7GdSZ	~1.4–1.5	10.5–11.5	200–250	Lower thermal conductivity, improved stability	Medium	Good
5Gd1Yb-YSZ	~1.7	10.5–11.5	200–250	Improved toughness & stability	Medium	Good
GYb-YSZ	<2	10.5–11.5	200–250	High-temperature stability	Medium–High	Good
Gd_2_Zr_2_O_7_	~1.5	~9	150–200	Low thermal conductivity, limited toughness	High	Promising
(Gd0.9Yb0.1)_2_Zr_2_O_7_	~1.4–1.5	9–10	150–200	Strong CMAS barrier	High	Good
La_2_Ce_2_O_7_	~1.6	9–10	150–200	Sintering resistant	High	Good
High-entropy oxides (YbGdTaHfZr)	0.61–0.89	~11.1	150–200	Ultra-low thermal conductivity	High	High potential
High-entropy RE zirconates	~1.3	10–11	150–200	Stable structure	High	High potential

**Table 2:** Comparison of Typical Bond Coat Systems for Thermal Barrier Coatings (TBCs) [54,55,56,57,58,59,60,61,62,63,64,65,66,67,68,69,70,71,72,73,74].

Bond Coat Type	Typical Composition	Typical Thickness (μm)	Main Advantages	Main Limitations	Deposition Methods
MCrAlY	NiCoCrAlY, CoNiCrAlY	100–300	Excellent oxidation resistance, good CTE match, mature technology	Al depletion during long-term exposure	APS, HVOF, LPPS
Pt-modified aluminide	Pt-Al	30–100	Superior TGO adhesion and oxidation resistance	High cost, brittle	Electroplating + aluminizing
High-entropy bond coat	Multi-principal alloy systems	50–150	Improved oxidation resistance, high-temperature stability	Limited long-term service validation	HVOF, laser cladding

**Table 3:** Typical Bond-Layer and Interlayer Systems in Environmental Barrier Coatings [75,76,77,78,79,80,81,82,83,84,85].

Layer Type	Typical Material	Typical Thickness (μm)	Main Function	Main Advantages	Main Limitations
Si bond layer	Si	50–150	Oxidation protection and adhesion	Mature technology, good oxidation resistance	SiO_2_ formation & volatilization at high T
Mullite interlayer	3Al_2_O_3_·2SiO_2_	50–200	Mitigate thermal expansion mismatch	Good CTE match	Crack-prone
BSAS layer	BaO–SrO–Al_2_O_3_–SiO_2_	100–300	Enhance water vapor stability	Good environmental stability	Reactivity with Si
Rare-earth silicate layer	Yb_2_Si_2_O_7_, Y_2_Si_2_O_7_, etc.	100–300	High-temperature water vapor corrosion resistance	Excellent high-T stability	CMAS sensitive
Multi-layer EBC	Si + Mullite + RE silicate	200–500	Integrated protection	Current mainstream structure	Complex fabrication

**Table 4:** Typical EBC Top-Coat Materials and Their Service Performance [86,87,88,89,90,91,92,93,94,95,96,97,98,99,100,101,102,103].

Material	Thermal Conductivity (W·m^−1^·K^−1^)	CTE (10^−6^ K^−1^)	Typical Thickness (μm)	Main Advantages & Limitations	CMAS Resistance	Engineering Evaluation
BSAS	~1.2–1.4	7–8	100–150	Good water vapor stability; Ba volatilization	Medium	Medium temperature applications
RE_2_SiO_5_	~1.1–1.3	6–8	100–150	Excellent chemical stability	High	Good
RE_2_Si_2_O_7_	~1.0–1.2	6–7	100–150	Good CTE match	High	Good
Yb_2_Si_2_O_7_	~1.1	6–7	100–150	High-temperature stability	High	High potential
High-entropy RE silicates	~1.0	6–7	100–150	Single-phase stability	High	High potential
β-(Er,Tm,Yb,Lu)_2_Si_2_O_7_	~1.0	6–7	100–150	Excellent water vapor resistance	High	High potential
Hf_6_Ta_2_O_17_	~1.2	6–7	80–120	Strong CMAS barrier	High	High temperature applications
Yb_2_SiO_5_	~1.1	6–7	100–150	Water/oxygen corrosion resistant	High	Good

## Data Availability

The original contributions presented in this study are included in the article. Further inquiries can be directed to the corresponding author.

## Abbreviations

Abbreviation	Full Name
APS	Atmospheric Plasma Spraying
BC	Bond Coat
BSAS	Barium-Strontium-Aluminum-Silicate
CMAS	Calcium-Magnesium-Aluminosilicate
CMC	Ceramic Matrix Composite
CTE	Coefficient of Thermal Expansion
DFT	Density Functional Theory
EB-PVD	Electron Beam-Physical Vapor Deposition
EBC	Environmental Barrier Coating
EDS	Energy Dispersive Spectroscopy
GZO	Gadolinium Zirconate (Gd_2_Zr_2_O_7_)
GYAG	(Gd,Y)_3_Al_5_O_12_
HEA	High-Entropy Alloy
HVAF	High-Velocity Air Fuel
HVOF	High-Velocity Oxygen Fuel
ICME	Integrated Computational Materials Engineering
MCrAlY	Metal-Chromium-Aluminum-Yttrium (M = Ni and/or Co)
MSZ	Multi-component rare-earth oxide-modified zirconia
NYSZ	Nanostructured Yttria-Stabilized Zirconia
RE	Rare Earth
SEM	Scanning Electron Microscopy
TBC	Thermal Barrier Coating
TCP	Topologically Close-Packed
TEM	Transmission Electron Microscopy
TGO	Thermally Grown Oxide
YSZ	Yttria-Stabilized Zirconia
